# Evaluating the risk of osteoporosis-related adverse events with proton pump inhibitors: a pharmacovigilance study

**DOI:** 10.3389/fphar.2025.1582908

**Published:** 2025-07-11

**Authors:** Jingkai Di, Nan Yang, Yicong Zhao, Shuai Chen, Zijian Guo, Dongdong He, Chuan Xiang

**Affiliations:** ^1^ Department of Orthopedics, Second Hospital of Shanxi Medical University, Taiyuan, China; ^2^ Shanxi Key Laboratory of Bone and Soft Tissue Injury Repair, The Second Hospital of Shanxi Medical University, Taiyuan, China; ^3^ School of Clinical Medicine, The Second Affiliated Hospital of Shanxi Medical University, Taiyuan, China; ^4^ Department of Orthopedics, The Third Hospital of Shanxi Medical University, Shanxi Bethune Hospital, Shanxi Academy of Medical Sciences Tongji Shanxi Hospital, Taiyuan, Shanxi, China; ^5^ Department of Rheumatology, The Second Hospital of Shanxi Medical University, Taiyuan, China

**Keywords:** proton pump inhibitors, osteoporosis, FAERS, adverse drug events, drug safety

## Abstract

**Background:**

Proton pump inhibitors (PPIs) are the most effective antacids and are widely used in the treatment of acid-related diseases. However, the impact of PPIs on bone remains controversial. This study aimed to explore the association between PPIs and osteoporosis-related adverse events in the real world.

**Materials and methods:**

Data from the United States Food and Drug Administration Adverse Event Reporting System from the first quarter of 2004 to the third quarter of 2024 were included in this study. Four pharmacovigilance analyses, reporting odds ratio (ROR), proportional reporting ratio (PRR), information component (IC), and Empirical Bayes geometric mean (EBGM) were used to explore the association between PPIs use and osteoporosis-related adverse events. In addition, we used the Bonferroni corrected P values and 95% confidence interval (95%CI). Meanwhile, the situation of different age and gender groups was examined using subgroup analysis. Additionally, evoked times and Weibull distributions were used to analyze the data further.

**Results:**

At the Primary terms level, esomeprazole, omeprazole and pantoprazole were found to have positive adverse event signals. However, at the overall dimension level of Standardized Medical Dictionary for Regulatory Activities (MedDRA) query, only esomeprazole (ROR: 8.83, 95%CI: 8.53–9.13, P < 0.001) and omeprazole (ROR: 1.54, 95%CI: 1.44–1.66, P < 0.001) signals were positive. Based on subgroup stratification, the study showed that the signal intensity of adverse events was stronger among women and older adults. Weibull distribution analysis indicated that the incidence of osteoporosis-related adverse events of esomeprazole increased gradually over time, while the risk of omeprazole did not show regular spatial and temporal distribution.

**Conclusion:**

This study comprehensively reports the risk of osteoporosis in the clinical use of five commonly used PPIs, which provides certain ideas and insights for the clinical prevention of such adverse events.

## 1 Introduction

Proton pump inhibitors (PPIs), a class of benzimidazole derivatives, are mainly used for the treatment of acid-digestive disorders, accounting for about 95% of prescriptions for acid-suppressive drugs (McGowan et al., 2010). PPIs play a role by irreversibly inhibiting the H+/K+ atpase in gastric parietal cells, thereby blocking the secretion of gastric acid (Savarino et al., 2009; Sun et al., 2024). Currently, PPIs have been widely used in the treatment of all known acid-related diseases, such as gastroesophageal reflux disease, non-steroidal anti-inflammatory drugs-induced gastrointestinal lesions, peptic ulcer, and *Helicobacter pylori* infection. (McGowan et al., 2010).

Given the remarkable efficacy of PPIs in the treatment of gastric acid-related disorders, patients tend to choose to use these drugs for a longer period. In England, about a quarter of patients taking PPIs continue to take them for more than a year (Farrell et al., 2022). The impact of long-term use of certain drugs (such as PPIs) on bone health, particularly the risk of osteoporosis, is controversial. Large epidemiological studies, such as those in Denmark and the UK, suggest that high-dose PPI use may increase the risk of hip fractures. (Savarino et al., 2009). However, other studies have suggested that no significant differences in bone mineral density or bone turnover markers between long-term PPI users and non-users (Targownik et al., 2017). The divergent results may be due to differences in study designs and populations, so the effect of PPIs on bone health remains a topic of ongoing debate.

Among the five clinically used PPIs (omeprazole, esomeprazole, lansoprazole, pantoprazole, rabeprazole), structural differences in side chains lead to divergent pharmacokinetic and safety profiles (Lespessailles and Toumi, 2022). Therefore, individualized selection of appropriate PPIs can not only effectively promote the remission of the disease but also reduce the occurrence of adverse reactions. With the development of population aging, the change of Bone Mineral Density (BMD) has become an important consideration when choosing an appropriate PPI, especially for women and the older (Ayers et al., 2023). Research shows that the effects of PPI on BMD and fracture risk are not consistent, for example, rabeprazole and esomeprazole may be associated with higher fracture risk, while omeprazole and pantoprazole are more likely to cause hypomagnesemia, which is a key cause of loss of BMD (van der Hoorn et al., 2015; Lespessailles and Toumi, 2022; Zeng et al., 2022). Therefore, it is urgent to clarify the effects of different PPIs on BMD.

The United States Food and Drug Administration Adverse Event Reporting System (FAERS) is a global largest publicly accessible database that contains many voluntary adverse event reports submitted by consumers, manufacturers, healthcare professionals, and other stakeholders. It is mainly used to document adverse drug events and support post-marketing drug safety monitoring. In addition, data mining of drug-related case reports from spontaneous reporting systems can provide us with a valuable source of information about the real-life safety of a particular drug.

Therefore, through pharmacovigilance analysis based on FAERS database, this study aimed to clarify the association between PPIs and osteoporosis in the real world and provide new insights to fully understand their safety and drug regulation. In addition, by comparing the effects of different PPIs on osteoporosis, it is aimed to provide new ideas for the selection of different PPIs.

## 2 Materials and methods

### 2.1 Data sources

The data from the open-source United States Food and Drug Administration Adverse Event Reporting System (FAERS) from the first quarter of 2004 to the third quarter of 2024 were included in this study, and the pharmacovigilance study was conducted. Seven subset files were covered, including patient demographic and management information (DEMO), DRUG information (DRUG), adverse event coding (REAC), Patient outcome (OUTC), source of report (RPSR), start and end dates of treatment (THER), and indication for administration (INDI). These files are linked to relevant adverse events through PRIMARYID, CASEID, and drug_seq to construct a canonical association mechanism. In addition, the International Guidelines for Safety Reporting specification issued by the International Conference on Harmonization was also used for dynamic data adjustment, so as to establish a standardized reference system to realize the overall optimization of data in the adaptation process. It is worth noting that the quantity in Table 1 refers to the number of patients who have experienced osteoporosis events. And a patient may experience multiple adverse events.

**TABLE 1:** Basic information about five proton pump inhibitors and osteoporosis events.

Characteristics	Total-all PPIs		Esomeprazole		Omeprazole		Lansoprazole		Rabeprazole		Pantoprazole	
Adverse events	total	SMQ-osteoporosis	total	SMQ-osteoporosis	total	SMQ-osteoporosis	total	SMQ-osteoporosis	total	SMQ-osteoporosis	total	SMQ-osteoporosis
N	210760	3702	66022	2766	77162	693	33268	80	3920	20	30388	143
Sex, n%												
Female	104928 (49.8%)	2293 (61.9%)	37608 (57.0%)	1642 (59.4%)	38794 (50.3%)	476 (68.7%)	11599 (34.9%)	54 (67.5%)	1804 (46.0%)	15 (75.0%)	15123 (49.8%)	106 (74.1%)
Male	63600 (30.2%)	664 (17.9%)	19073 (28.9%)	476 (17.2%)	25045 (32.5%)	134 (19.3%)	7591 (22.8%)	20 (25.0%)	1168 (29.8%)	5 (25.0%)	10723 (35.3%)	29 (20.3%)
Unknown	42232 (20.0%)	745 (20.1%)	9341 (14.1%)	648 (23.4%)	13323 (17.3%)	83 (12.0%)	14078 (42.3%)	6 (7.5%)	948 (24.2%)	0 (0%)	4542 (14.9%)	8 (5.6%)
Age, n%												
<18	2638 (1.3%)	5 (0.1%)	482 (0.7%)	3 (0.1%)	1357 (1.8%)	1 (0.1%)	517 (1.6%)	1 (1.3%)	21 (0.5%)	0 (0%)	261 (0.9%)	0 (0%)
>85	5190 (2.5%)	50 (1.4%)	1179 (1.8%)	23 (0.8%)	1850 (2.4%)	18 (2.6%)	934 (2.8%)	5 (6.3%)	154 (3.9%)	1 (5.0%)	1073 (3.5%)	3 (2.1%)
18–64.9	60196 (28.6%)	1478 (57.0%)	21550 (32.6%)	1141 (41.3%)	21371 (27.7%)	242 (34.9%)	5413 (16.3%)	24 (30.0%)	1242 (31.7%)	5 (25.0%)	10620 (34.9%)	66 (46.2%)
65–85	40365 (19.2%)	628 (39.9%)	11076 (16.8%)	388 (14.0%)	15659 (20.3%)	165 (23.8%)	5003 (15.0%)	30 (37.5%)	971 (24.8%)	7 (35.0%)	7656 (25.2%)	38 (26.6%)
Unknow	102371 (48.6%)	1541 (41.6%)	31735 (48.1%)	1211 (43.8%)	36925 (47.9%)	267 (38.5%)	21401 (64.3%)	20 (25.0%)	1532 (39.1%)	7 (35.0%)	10778 (35.5%)	36 (25.2%)
Outcome												
CA	358 (0.2%)	0 (0%)	38 (0.1%)	0 (0%)	182 (0.2%)	0 (0%)	28 (0.1%)	0 (0%)	4 (0.1%)	0 (0%)	106 (0.3%)	0 (0%)
DE	13364 (6.3%)	62 (1.7%)	3415 (5.2%)	47 (1.7%)	4634 (6.0%)	6 (0.9%)	2900 (8.7%)	2 (2.5%)	373 (9.5%)	0 (0%)	2042 (6.7%)	7 (4.9%)
DS	3274 (1.6%)	328 (8.9%)	925 (1.4%)	249 (9.0%)	1524 (2.0%)	65 (9.4%)	381 (1.1%)	3 (3.8%)	70 (1.8%)	0 (0%)	374 (1.2%)	11 (7.7%)
HO	37612 (17.8%)	1040 (28.1%)	9815 (14.9%)	807 (29.2%)	14809 (19.2%)	139 (20.1%)	4696 (14.1%)	27 (33.8%)	1084 (27.7%)	9 (45.0%)	7208 (23.7%)	58 (40.6%)
LT	4887 (2.3%)	25 (0.7%)	791 (1.2%)	6 (0.2%)	2315 (3.0%)	18 (2.6%)	623 (1.9%)	0 (0%)	95 (2.4%)	0 (0%)	1063 (3.5%)	1 (0.7%)
OT	89854 (42.6%)	633 (17.1%)	22202 (33.6%)	353 (12.8%)	31674 (41.0%)	201 (29.0%)	19351 (58.2%)	31 (38.8%)	1386 (35.4%)	6 (30.0%)	15241 (50.2%)	42 (29.4%)
RI	549 (0.3%)	5 (0.1%)	132 (0.2%)	1 (0.0%)	116 (0.2%)	0 (0%)	163 (0.5%)	2 (2.5%)	98 (2.5%)	0 (0%)	40 (0.1%)	2 (1.4%)
Unknown	60862 (28.9%)	1609 (43.5%)	28704 (43.5%)	1303 (47.1%)	21908 (28.4%)	264 (38.1%)	5126 (15.4%)	15 (18.8%)	810 (20.7%)	5 (25.0%)	4314 (14.2%)	22 (15.4%)

Abbreviations: N: the number of patients who have experienced osteoporosis related adverse events, CA: congenital anomaly, DE: death, DS: disability, HO: Hospitalization-Initial or Prolonged, LT: Life-Threatening, OT: other serious important medical event, RI: required intervention to prevent permanent.

### 2.2 Standardized definition of adverse events

All adverse events were coded using preferred terms (PT) according to the International Medical Dictionary of Regulatory Activities (MedDRA) (version 26.1). To improve data accuracy and standardization, the study introduced a Standardized Medical Dictionary for Regulatory Activities query (SMQ). The broad and narrow definition of SMQ is a rule set for the classification and retrieval of medical concepts. The narrow definition is applied to the retrieval of “20000178: osteoporosis/osteopenia” adverse events were sorted and classified to match the spontaneously reported adverse events with relevant definitions. Specifically, a total of ten preferred terms were included in SMQ ″20000178: osteoporosis/osteopenia”, which were “10049470: Bone density decreased”, “10056809: Bone formation decreased”, “10065687: Bone loss”, “10064269: Bone marrow edema syndrome”, “10049088: Osteopenia”, “10031282: Osteoporosis”, “10031285: Osteoporosis postmenopausal”, “10031290: Osteoporotic fracture”, “10038642: Bone resorption increased” and “10039984: Senile osteoporosis”. In this study, “SMQ-osteoporosis” represents all adverse events related to osteoporosis.

### 2.3 Data preparation and procedures

Firstly, this study used MeSH titles to query the common names and trade names of five PPIs, and extracted relevant adverse event reports from the FAERS database. In addition, the study deeply cleaned the original data to identify and eliminate recording errors, missing key data, and duplicate and redundant reports, aiming to minimize data bias and improve the value of in-depth data mining. Specifically, when CASEID is the same, the latest FDA_DT report will be selected; When FDA_DT and CASEID are the same, choose the higher PRIMARYID report (Liu R. et al., 2024). This method ensures that only the most accurate and up-to-date reports are included. In addition, this study excluded reports lacking key demographic data or related to unapproved uses to reduce potential bias. The PTs criteria for MedDRA terminology adverse effect classification classified the drugs into four patterns: PS (primary suspicion), SS (second suspicion), C (concomitant effect), and I (interaction). To ensure that drug adverse events are highly suspected to be caused by the drug itself. This study focuses on reports where the role code of the drug in the document is “PS”. In addition, this study sorted out the proportion of cases with serious consequences caused by PPIs and the onset time data of PT involved in the corresponding signal, so as to deepen the analysis of the safety characteristics of this class of drugs.

### 2.4 Statistical analysis

Disproportion analysis as a method of signal detection is used in this paper in the field of pharmacovigilance. Specifically, when defining and identifying adverse events (AEs), the study used four methods: reporting odds ratio (ROR), proportional reporting ratio (PRR), information component (IC), and Empirical Bayes geometric mean (EBGM). The criteria for a positive signal in the ROR method include having three or more reports and a lower limit of the 95% confidence interval (CI) with an ROR greater than 1. Similarly, the PRR method include having three or more reports, a PRR greater than or equal to 2, and a chi square value greater than or equal to 4. The IC method adopts a positive signal detection standard, where the lower limit (IC025) of the 95% CI needs to be greater than 0. Finally, the EBGM method uses positive signal detection criteria, where the lower limit of the 95% confidence interval (EBGM05) needs to be greater than two and the number of cases needs to be greater than 0 (Gao et al., 2025). In order to identify all potential drug safety hazards, according to the pharmacovigilance information standard, the report that meets any of the above four methods and achieves a positive signal value is regarded as a positive adverse event report. The corresponding specific calculation formula is listed in Supplementary Table S1 (Rothman et al., 2004). In addition, the study used P-values based on chi-square tests to further validate the statistical significance of the results. The calculation of P-value was independent and mutually validated with the four pharmacovigilance analysis methods. In order to decrease the occurrence rate of type I error caused by multiple comparisons and improve the accuracy and reliability of the research results, we adjusted the P-value using the Bonferroni method. In addition, we conducted subgroup analyses stratified by age and gender to reduce the influence of confounding variables. In the subgroup analysis based on age, 60 years old was the criterion to divide the population into the younger group and the older group. In addition, the Weibull distribution analysis method was used to dynamically analyze the evolution trajectory of the occurrence of adverse events from the time dimension to expand the research horizon. Based on the shape parameter β and its 95% confidence interval (CI), the risk in a reference population can be assessed, categorized as follows: when β < 1 and its 95% CI < 1, it is considered that the risk of drug-related AEs decreases over time (early failure type); when β is equal to or close to one and its 95% CI includes 1, it is considered that the risk of drug-related AEs occurs with no specific spatiotemporal pattern (random failure type); when β > 1 and its 95% CI > 1, it is considered that the risk of drug-related AEs increases over time (wear-out failure type) (Liu Y. et al., 2024). R 4.4.2 and its RStudio were also used to ensure efficient, accurate and reproducible analysis.

## 3 Results

### 3.1 Descriptive analysis

**FIGURE 1:**
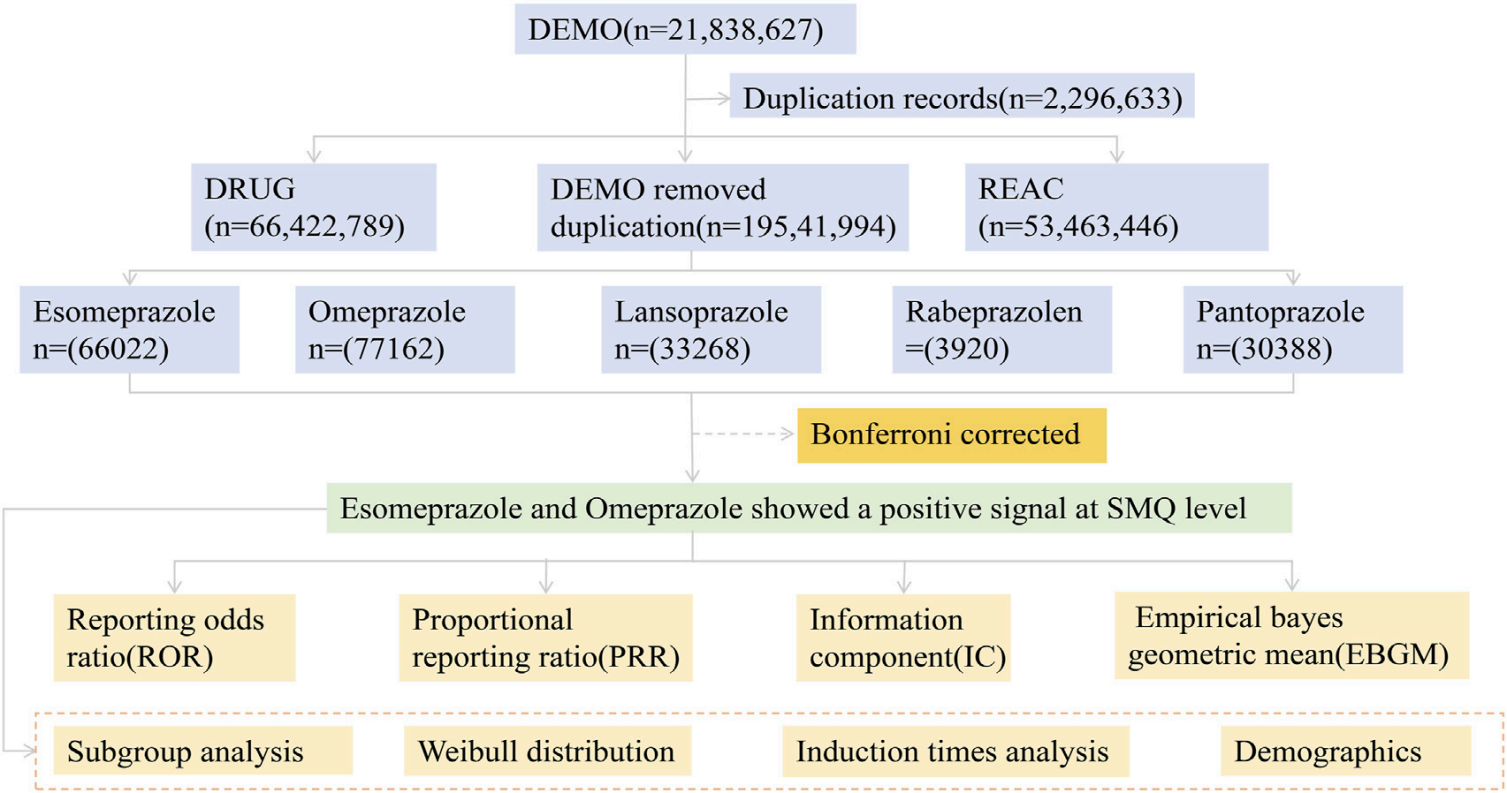
Flow chart showing the analysis process of the study.

During the testing period from the first quarter of 2004 to the third quarter of 2024, 210,760 adverse event reports of PPIs were recorded in the FAERS database. Of these, 3,702 were related to osteoporosis events (Figure 1). The basic information of patients taking five PPIs is given in Table 1. Esomeprazole was reported most frequently (n = 2,766, 74.7%) and rabeprazole was reported least frequently (n = 20, 0.5%). In the reports of adverse events caused by PPIs, the proportion of female patients (n = 104928, 49.8%) was higher than that of male patients (n = 63,600, 30.2%). Of note, when specific to osteoporosis events, the number of female patients was approximately three times higher (n = 2,293, 61.9%) than that of male patients (n = 664, 17.9%). When paying attention to the age composition of patients, PPIs-related osteoporosis events were more common in those aged 18–65 years. (n = 1,478, 39.9%). In addition, hospitalization (n = 1,040, 28.1%) and other serious adverse events (n = 633, 17.1%) accounted for a greater proportion of the outcomes in patients who developed osteoporosis while taking PPIs.

### 3.2 Signal mining

**FIGURE 2:**
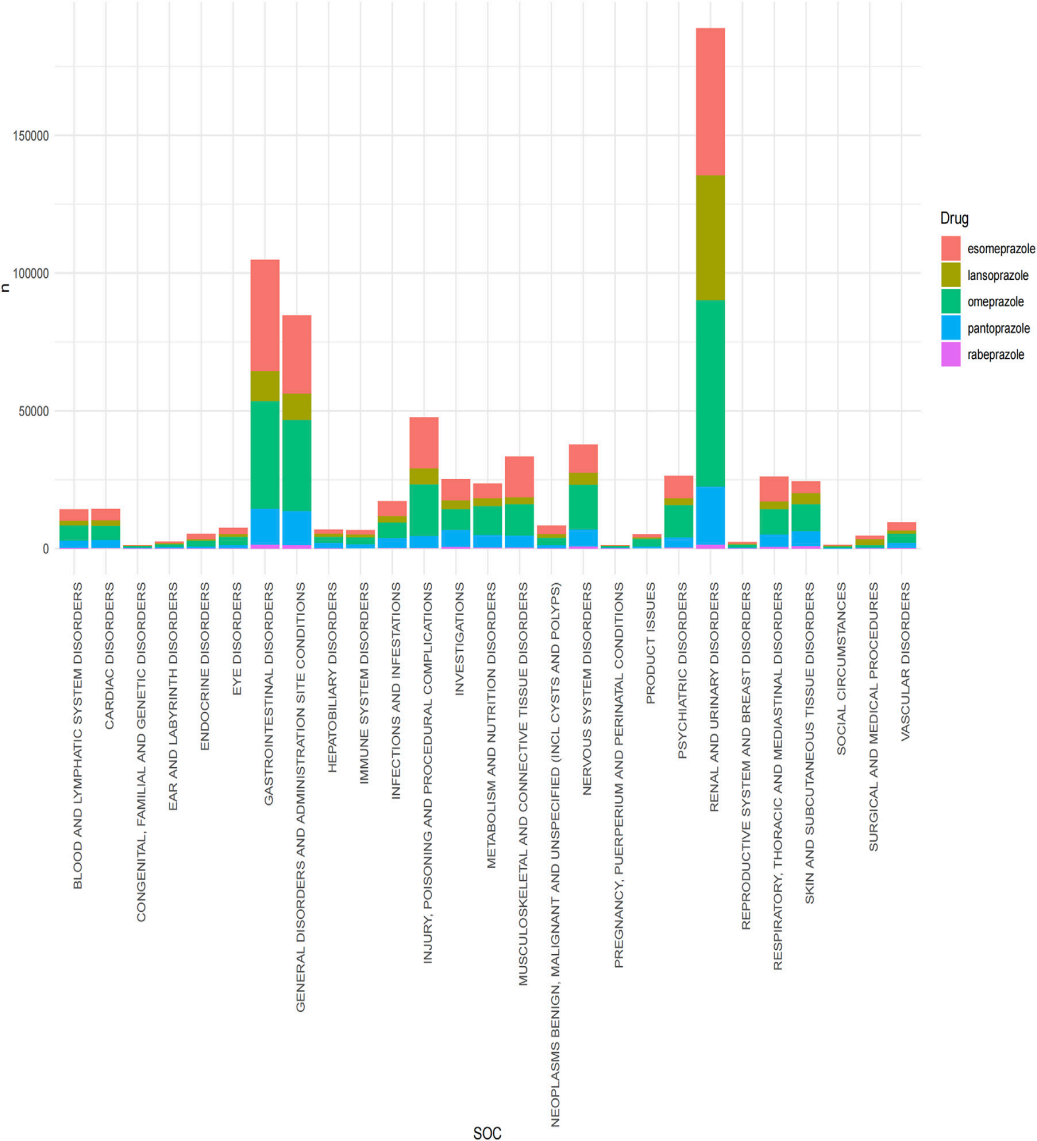
Signal detection of five proton pump inhibitors at the system organ level (SOC). The results are presented using a bar chart. Different colors represent different drugs, and the height of the bar chart represents the quantity.

Statistical analysis revealed that a total of 27 organ systems were affected by PPIs-related adverse events at the SOC level (Figure 2). Among them, the most affected systems are renal and urinary disorders (n = 188941). gastrointestinal disorders were also common. (n = 104787) while pregnancy, puerperium, and perinatal conditions accounted for the least number of SOC. (n = 1189) In terms of signal intensity, renal and urinary disorders showed the strongest positive signal among the five PPIs. In particular, the signal was the strongest in lansoprazole (ROR: 39.84, 95%CI: 39.35–40.33). (Supplementary Table S2).

**FIGURE 3:**
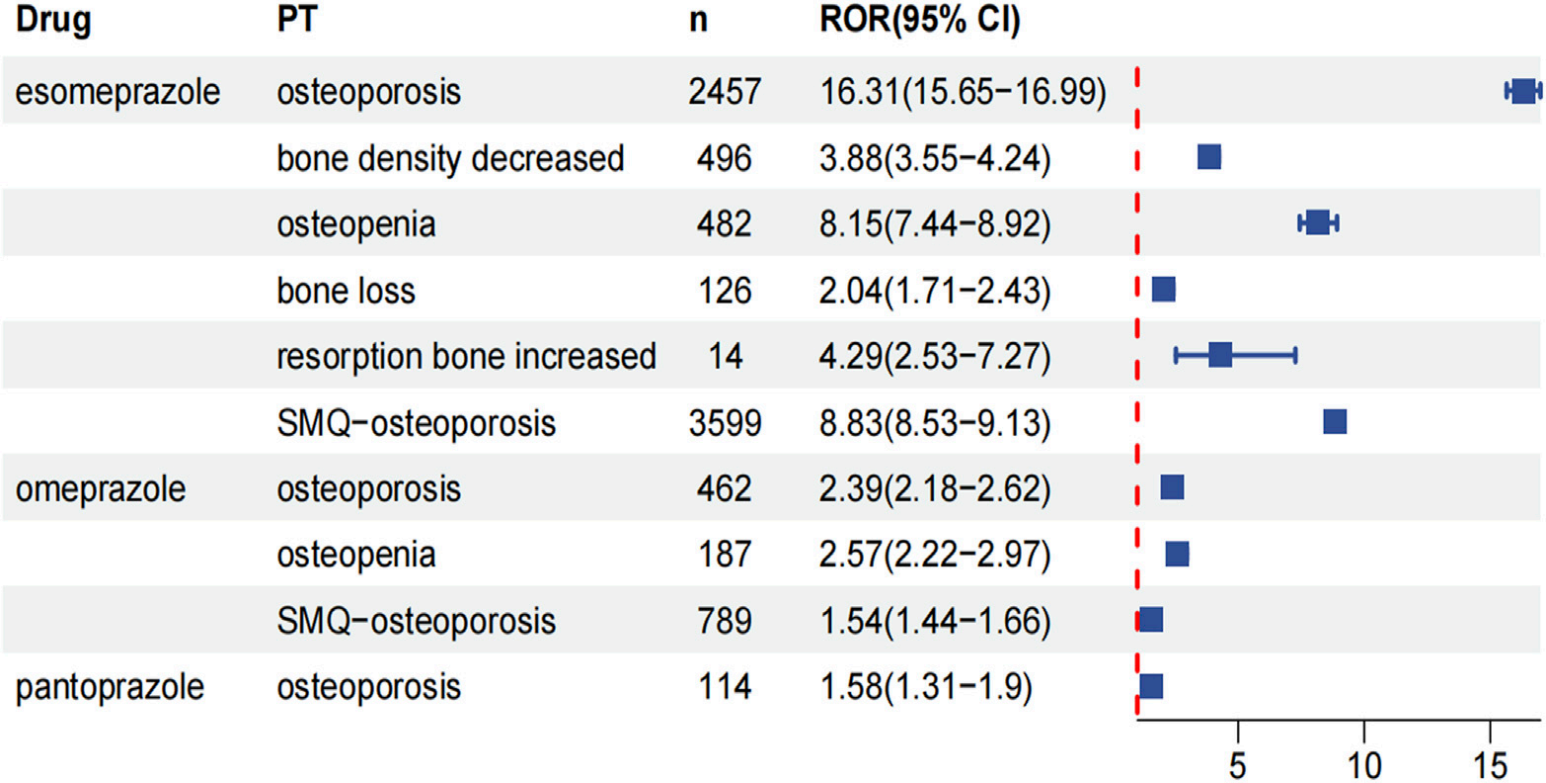
Forest plots of the ROR and their corresponding 95% CI for five PPIs at the PT level. The value of “n” represents the number of adverse events related to osteoporosis.

When focusing on the PT level, only three drugs (esomeprazole, omeprazole, and pantoprazole) were retrieved with positive signals (Figure 3). Esomeprazole had the highest number of osteoporosis events (n = 3599), including five positive PT. Osteoporosis was the most common adverse event of osteoporosis with the highest positive signal value (ROR: 16.31, 95%CI: 15.65–16.99, P < 0.001). In addition, a high positive signal was also found for osteopenia (ROR: 8.15, 95%CI: PT7.44-8.92, P < 0.001). Second, two positive PTs were retrieved in 789 osteoporotic events associated with omeprazole. Osteoporosis (ROR: 2.39, 95%CI: 2.39, P < 0.001) and osteopenia (ROR: 2.57, 95%CI: 2.22–2.97, P < 0.001). Pantoprazole only detected a positive signal of osteoporosis (ROR: 1.58, 95%CI: 1.31–1.9, P < 0.001). Of note, rabeprazole and lansoprazole were not retrieved for associated positive events.

In addition, SMQ contains a related set of PTs with a more stringent screening and integration mechanism. Therefore, the SMQ analysis was used to further reflect the overall association between drugs and osteoporosis events. The results showed that only two drugs showed positive signals: esomeprazole (ROR: 8.83, 95%CI: 8.53–9.13, P < 0.001) and omeprazole (ROR: 1.54, 95%CI: 1.44–1.66, P < 0.001). Of note, although pantoprazole was associated with osteoporotic events at the PT level, no positive signal was retrieved when its global signal was evaluated.

Furthermore, subgroup analysis of the two drugs that still had a positive signal on the global domain of the SMQ showed that the signal intensity of osteoporosis-related adverse events was stronger in women than in men. The results of subgroup analysis are shown in Table 2. Specifically, among patients taking esomeprazole, the overall reported pharmacovigilance signal was stronger in women (ROR: 9.47, 95%CI: 9.07–9.89, P < 0.001) than in men (ROR: 4.41, 95%CI: 4.07–4.77, P < 0.001). Similarly, in the omeprazole group, the signal value of females (ROR: 2.19, 95%CI: 2.02–2.38, P < 0.001) was stronger than that of males (ROR: 0.74, 95%CI: 0.63–0.87, P < 0.001). At the same time, when focusing on the PT dimension, it was found that osteoporosis was the only adverse event that presented positive signals in both subgroups of the two classes of drugs. Based on this common event analysis, the study showed that the signal value of adverse events in women taking esomeprazole (ROR: 13.67, 95%CI: 12.96–14.43, P < 0.001) was still higher than that in men taking esomeprazole (ROR: 10.80, 95%CI: 9.79–11.92, P < 0.001). The same outcomes were observed within the omeprazole group.

**TABLE 2:** Subgroup analysis of esomeprazole and omeprazole based on age and gender.

Drug	Subgroup	PT	N	ROR (95%Cl)	PRR (χ^2^)	EBGM (EBGM05)	IC (IC025)	P Value
Esomeprazole	Younger	osteoporosis	891	18.91 (17.66–20.25)	18.69 (13902.73)	17.47 (16.50)	4.13 (4.03)	<0.001
		osteopenia	220	8.99 (7.86–10.28)	8.97 (1503.95)	8.69 (7.77)	3.12 (2.92)	<0.001
		bone density decreased	137	2.41 (2.03–2.85)	2.41 (111.55)	2.39 (2.08)	1.26 (1.01)	<0.001
		senile osteoporosis	2	31.55 (7.25–137.21)	31.55 (52.58)	28.15 (8.23)	4.82 (3.02)	<0.05
		SMQ-osteoporosis	1309	8.24 (7.80–8.71)	8.12 (7933.30)	7.9 (7.54)	2.98 (2.90)	<0.001
	Older	osteoporosis	506	11.55 (10.56–12.64)	11.48 (4603.48)	10.96 (10.17)	3.45 (3.32)	<0.001
		osteopenia	177	12.38 (10.64–14.41)	12.35 (1749.91)	11.75 (10.35)	3.56 (3.33)	<0.001
		bone density decreased	83	4.03 (3.24–5.01)	4.03 (185.41)	3.97 (3.31)	1.99 (1.67)	<0.001
		bone loss	34	4.02 (2.86–5.64)	4.02 (75.70)	3.96 (2.98)	1.99 (1.49)	<0.001
		resorption bone increased	10	7.08 (3.77–13.3)	7.08 (50.62)	6.89 (4.07)	2.79 (1.90)	<0.001
		SMQ-osteoporosis	817	8.94 (8.33–9.59)	8.84 (5471.05)	8.54 (8.05)	3.09 (2.99)	<0.001
	Male	osteoporosis	415	10.8 (9.79–11.92)	10.74 (3528.25)	10.37 (9.55)	3.37 (3.23)	<0.001
		bone density decreased	100	1.76 (1.45–2.15)	1.76 (32.80)	1.76 (1.49)	0.81 (0.53)	<0.001
		osteopenia	83	4.38 (3.53–5.45)	4.38 (213.10)	4.33 (3.61)	2.11 (1.80)	<0.001
		resorption bone increased	6	10.86 (4.8–24.56)	10.86 (51.65)	10.48 (5.29)	3.39 (2.27)	<0.001
		SMQ-osteoporosis	639	4.41 (4.07–4.77)	4.37 (1639.77)	4.32 (4.04)	2.11 (2.00)	<0.001
	Female	osteoporosis	1426	13.67 (12.96–14.43)	13.55 (15571.34)	12.78 (12.22)	3.68 (3.60)	<0.001
		osteopenia	388	10.49 (9.47–11.61)	10.46 (3162.18)	10.01 (9.19)	3.32 (3.17)	<0.001
		bone density decreased	260	4.60 (4.07–5.21)	4.6 (716.19)	4.52 (4.08)	2.18 (2.00)	<0.001
		bone loss	84	3.30 (2.66–4.10)	3.3 (132.80)	3.27 (2.73)	1.71 (1.39)	<0.001
		senile osteoporosis	4	8.78 (3.23–23.87)	8.78 (26.45)	8.46 (3.66)	3.08 (1.76)	<0.05
		SMQ-osteoporosis	2185	9.47 (9.07–9.89)	9.34 (15597.15)	8.98 (8.66)	3.17 (3.10)	<0.001
Omeprazole	Younger	osteoporosis	115	2.14 (1.78–2.57)	2.14 (69.20)	2.13 (1.83)	1.09 (0.82)	<0.001
		osteopenia	69	2.59 (2.04–3.29)	2.59 (66.69)	2.57 (2.11)	1.36 (1.02)	<0.001
		SMQ-osteoporosis	209	1.19 (1.04–1.37)	1.19 (6.40)	1.19 (1.06)	0.25 (0.05)	<0.001
	Older	osteoporosis	183	3.22 (2.78–3.72)	3.21 (273.72)	3.17 (2.81)	1.66 (1.45)	<0.001
		osteopenia	71	3.83 (3.03–4.85)	3.83 (145.50)	3.77 (3.10)	1.92 (1.57)	<0.001
		SMQ-osteoporosis	302	2.55 (2.28–2.86)	2.55 (280.24)	2.53 (2.30)	1.34 (1.17)	<0.001
	Male	osteoporosis	96	1.77 (1.45–2.16)	1.77 (31.86)	1.76 (1.49)	0.82 (0.52)	<0.001
		SMQ-osteoporosis	150	0.74 (0.63–0.87)	0.74 (13.17)	0.75 (0.65)	0.42 (−0.66)	<0.001
	Female	osteoporosis	315	2.71 (2.43–3.03)	2.71 (335.62)	2.69 (2.45)	1.43 (1.26)	<0.001
		osteopenia	150	3.74 (3.18–4.39)	3.73 (294.73)	3.68 (3.22)	1.88 (1.64)	<0.001
		SMQ-osteoporosis	556	2.19 (2.02–2.38)	2.19 (355.37)	2.18 (2.03)	1.12 (1.00)	<0.001

*Younger: Population under 60 years old. Older: Population over 60 years old. Abbreviations: N: the number of adverse events related to osteoporosis, ROR: report odds ratio, PRR: proportional reporting ratio, IC: information component, EBGM: Empirical Bayesian Geometric Mean and P value: Adjusted P value.

Subgroup analysis by age showed that the signal of adverse events related to osteoporosis was stronger in the older group than in the younger group. At the overall SMQ level, the signal value of adverse events was higher in the older group (ROR: 8.94, 95%CI: 8.33–9.59, P < 0.001) than in the young group (ROR: 8.24, 95%CI: 7.80–8.71, P < 0.001). This phenomenon is more prominent in the group taking omeprazole, with an ROR value of 2.55 (95% CI: 2.28–2.86; P < 0.001) in the older patients, significantly higher than the risk level of young patients (ROR = 1.19, 95% CI: 1.04–1.37; P < 0.001). When it comes to specific PT, the older patients treated with omeprazole exhibit stronger positive signals at each PT level. It is worth noting that in the signal detection of esomeprazole, the signal of osteoporosis as an adverse event in the young group (ROR = 18.91, 95% CI: 17.66–20.25, P < 0.001) was higher than that in the older group (ROR: 11.55, 95% CI: 10.56–12.64, P < 0.001). Except for this specific PT, the signal strength of other adverse events is still higher in the older population.

### 3.3 Time-to-onset analysis and weibull distribution analysis

**FIGURE 4:**
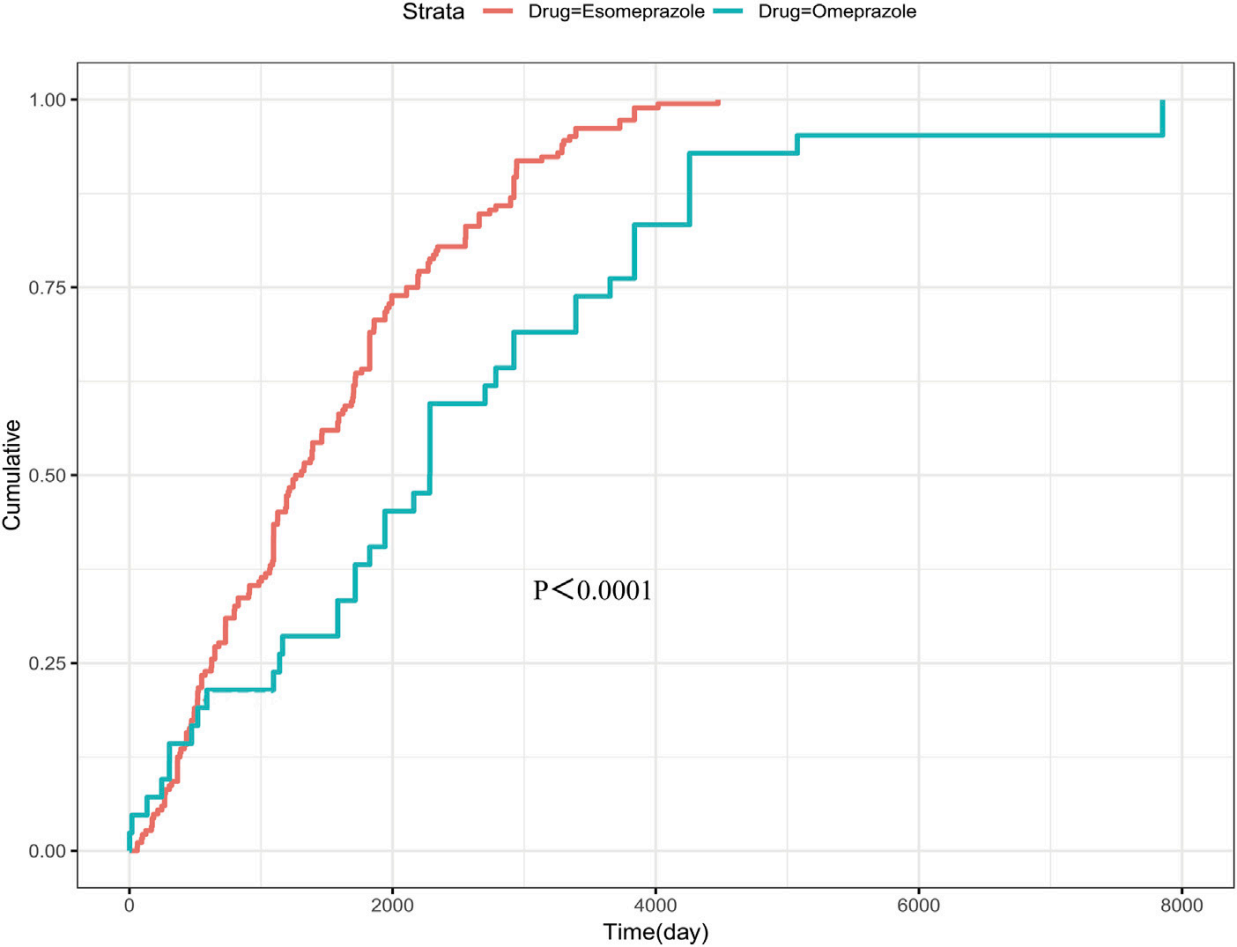
Survival curve of adverse events caused by esomeprazole and omeprazole. The p-value presented in the figure is derived from the log-rank test, which is used to assess the statistical significance of differences between the survival curves of these groups.

The study focused on esomeprazole and omeprazole in the assessment of the time to osteoporotic events after PPIs use. The average induction time of esomeprazole was about 1494.4 days, and the median induction time was about 1285.5 days. The AEs occurred as early as 59 days, but most of the AEs occurred more than 1 year later (about 88.0%). The mean induction time of omeprazole was about 2451.6 days, and the median induction time was about 2283 days. Similar to esomeprazole, most of the cases occurred after 1 year, accounting for about 85.7%. In addition, the adverse event survival plot showed a statistically significant difference in the time to induction of osteoporotic events between esomeprazole and omeprazole (P < 0.0001) (Figure 4). The Weibull distribution showed that the osteoporosis events caused by esomeprazole increased over time, showing a wear-out failure type. However, omeprazole showed a random failure type, indicating that the osteoporotic events induced by omeprazole have no spatiotemporal pattern. The results of the Weibull distribution are shown in Table 3.

**TABLE 3:** Shape parameters of Weibull distribution for esomeprazole and omeprazole.

Drug	Scale parameter: α	Shape parameter: β	Type
	95% CI	95% CI	
Esomeprazole	1650.50 (1480.50–1820.50)	1.48 (1.31–1.65)	Wear-out failure
Omeprazole	2535.35 (1827.53–3243.17)	1.12 (0.84–1.41)	Random Failure

## 4 Discussion

Based on the contradictory evidence of whether PPIs can cause osteoporosis events, the study is the first to use pharmacovigilance analysis to clarify the specific risk of PPIs on osteoporosis events in the real world, providing new insights for improving patient prognosis and clinical drug regulation. Research has shown that among the five commonly used PPIs, esomeprazole and omeprazole are associated with osteoporosis events in both overall and PT level signal assessments. However, the effect of pantoprazole on bone is relatively mild, and no positive signals were detected in the more rigorous overall SMQ assessment. However, rabeprazole and lansoprazole are not associated with the occurrence of osteoporosis events.

PPI can cause various osteoporosis events, which may be caused by multiple mechanisms Firstly, from the perspective of bone metabolism, gastric acid is an important condition for the absorption of insoluble calcium, and acid suppression leads to a decrease in the absorption of soluble calcium (Sheikh et al., 1987). While a negative calcium balance is a major cause of osteoporosis. In addition, long-term use of PPIs can have adverse effects on the absorption of vitamin B12, leading to muscle weakness and an increased risk of falls and fractures in the elderly (Lewis et al., 2014). In addition to its effects on calcium absorption and vitamins, PPIs can also lead to secondary hyperparathyroidism. This can lead to faster bone resorption than bone formation, resulting in decreased bone density. (Jansen et al., 1990).

In addition, more direct effects are elucidated. Specifically, an *in vitro* study suggesting that PPIs can reduce bone mass and increase the risk of osteoporotic events by inhibiting collagen production (Ghebre, 2020). Of note, a recent study based on Bhargavi V Desai et al. showed that PPIs can affect the process of bone remodeling by regulating TRPM7 channels in osteocytes (Desai et al., 2022). Additionally, drug-drug interactions and pre-existing diseases are also important influencing factors in the formation of osteoporosis-related adverse events. Based on the complexity of the main applicable population of PPIs, multi-drug combination regimens are often implemented. For example, it often require combined use of glucocorticoids (Heo, 2021; Dong et al., 2024). For example, glucocorticoids, as an important confounding factor, can lead to rapid reduction of bone mass, deterioration of bone microstructure, and decrease in bone strength through a multi-target mechanism. (Lane, 2019). In addition to the above additive effects, all five PPIs described in this paper were found to competitively inhibit the activity of CYP2C19, decreasing liver enzyme activity and slowing the metabolic rate of drugs such as glucocorticoids in the body, which may further increase the risk of osteoporosis-related adverse events (Li et al., 2004). It is important to acknowledge that baseline comorbidities may introduce a certain degree of confounding into the results of this study. But the existing evidence shows that such studies can still provide valuable insights for clinical practice. (Liu et al., 2025).

The differing effects of various PPIs on bone health may be attributed to variations in their drug metabolism and gastric pH regulation efficacy. Studies have shown that rabeprazole is rapidly metabolized in the liver in a non-enzymatic form, which makes it almost impossible to accumulate in the body, so its effect on osteoporosis events is minimal (Ishizaki and Horai, 1999). In addition, lansoprazole is metabolized via dual pathways (CYP2C19 and CYP3A4), avoiding toxic retention and conferring safety. However, compared with these two drugs, esomeprazole, omeprazole, and pantoprazole mainly rely on the CYP2C19 enzyme for metabolism, which may be the primary factor of these three drugs causing bone loss. Furthermore, pantoprazole was metabolized at a faster rate through this pathway than the other two drugs, which may be one explanation for its milder effects on bone (Yasuda et al., 1995; Li et al., 2004). It is noteworthy that esomeprazole exhibits significantly higher drug exposure and longer duration of action than omeprazole, attributed to its stronger and more sustained acid-suppressive effect and lower metabolic clearance rate (Andersson et al., 2001). These factors may lead to a higher pharmacovigilance adverse signal for esomeprazole compared with omeprazole (Sheikh et al., 1987).

The results in gender subgroups suggested that positive signals tended to be of higher intensity in women than in men. Differences in dose and duration of medication between populations may account for this result. A systematic review that integrated demographic and medication factors of PPIs users showed that 56% of PPIs users were women, nearly two-thirds of them tended to take high doses of PPIs, and 25% of PPIs users took PPIs for up to 1 year (Shanika et al., 2023). Additionally, conditional risk factors of medications may yield different outcomes due to gender disparities. Specifically, long-term use of PPIs exacerbates chronic atrophic gastritis in patients with *helicobacter pylori* infection, and females may be more susceptible to autoimmune-mediated gastric mucosal injury (Lahner et al., 2019). This, in turn, could result in poorer absorption of bone-nourishing factors and ultimately increase the risk of osteoporosis (Cavalcoli et al., 2017; Lahner et al., 2019). It is worth noting that menopause, as a unique period for women, is a key turning point for the occurrence of adverse events related to osteoporosis (Fischer and Haffner-Luntzer, 2022). During this period, female ovarian function declines, the sharp decrease in estrogen secretion leads to the relative increase of osteoclast activity, and the homeostasis of bone resorption and bone formation is unbalanced (de Villiers, 2024). Recent studies have shown that PPI use may aggravate this abnormal level of bone metabolism (da Maia et al., 2022).

The study showed higher pharmacovigilance signals for osteoporosis-related adverse events in the older population than in the younger group, and again, irrational medication patterns are one of the key effects. A retrospective analysis of PPI users older than 65 shows that PPIs are often used without appropriate indications or for longer than recommended durations (Mehta et al., 2020). In addition, older patients tend to take longer courses to achieve symptom relief than younger patients taking PPIs, with a median exposure of up to 4.6 years (Ben-Eltriki et al., 2020; Lechien, 2022). In addition, the liver drug enzyme activities in the older group were often worse than those in the younger group (Thürmann, 2020). This slows down the metabolic rate of PPIs in this special population, exacerbates the burden of drug exposure, and eventually leads to an increased risk of osteoporosis. It is worth noting that when focusing on the specific PT level, young patients taking esomeprazole have a higher positive signal for osteoporosis as an adverse event, which may be related to their poor lifestyle habits. Research suggests that irregular diet and bedtime dinner are risk factors for gastroesophageal reflux disease (Yamamichi et al., 2012). This may lead to a higher risk of gastroesophageal reflux disease in young people, thereby increasing the use of PPIs. In addition, a lack of exercise and other unhealthy lifestyle habits may indirectly increase the risk of osteoporosis (Zhang et al., 2022).

The results of Weibull distribution suggests that the pros and cons of esomeprazole treatment should be more carefully weighed according to the specific conditions of patients in the actual clinical process. For patients with mild disease who do not require long-term treatment with this drug, dose reduction or shorter treatment duration should be considered as soon as symptoms are controlled. For those who must use drugs for a long time, it is necessary to strengthen the monitoring and consider the preventive use of anti-osteoporosis drugs, so as to minimize the potential harm of adverse drug reactions and optimize the balance between benefit and risk of clinical treatment (Hant and Bolster, 2016).

The study has some limitations. Firstly, this study only used databases sourced from FAERS, and as a database based on spontaneous reporting characteristics, the FAERS database itself has the possibility of false positives. Secondly, based on the skill proficiency and autonomy tendency of the reporter, there is often an inherent selection bias. Especially for non-serious adverse events, there is a selective bias. In addition, the study did not quantify the risk and could not infer exact causal relationships, but only provided an estimate of signal strength. Therefore, higher-quality and larger-scale prospective studies are still needed to clarify the causal relationship and increase the credibility of current conclusions. On the other hand, the integrity of data has a certain impact on the results of subgroups. For example, the age of patients taking esomeprazole is unknown, resulting in significant data loss. Finally, the residual effects of potential confounding factors require careful consideration: for instance, the lack of systematic collection of information regarding comorbidities, concomitant medications, and prescription indications may exert a certain impact on the results. Therefore, further prospective studies are needed to validate this result before it can be applied to clinical practice.

## 5 Conclusion

The widespread use of PPIs in clinical practice has raised concerns about their safety, especially their potential for bone damage. Whether it can lead to osteoporosis events is the key controversy in clinical practice. Using multi-strategy real-world data mining analysis, an association between esomeprazole, omeprazole, and osteoporosis-related adverse events was found. This study helps to improve the understanding of the safety of PPIs, and also provides a valuable reference for the prevention of osteoporosis-related adverse events and clinical practice.

## Data Availability

Publicly available datasets were analyzed in this study. This data can be found here: FAERS database (https://www.fda.gov).
