# Supplementary material for: Evaluating the risk of osteoporosis-related adverse events with proton pump inhibitors: a pharmacovigilance study
Source: Front Pharmacol. 2025 Jul 11;16:1582908. doi: 10.3389/fphar.2025.1582908 (PMC12290295; doi:10.3389/fphar.2025.1582908)
Supplement: Supplementary file 1 [file DataSheet1.docx]

**Table S1** The formulas used for statistical testing.

| **Name of Algorithm** | **Formula** | **Positive Signal Criteria** |
| --- | --- | --- |
| **ROR** | ROR=ad/bc | Lower Limit of 95%CI>1, N≥3 |
|  | 95%CI=e ^ln(ROR)±1.96(1/a+1/b+1/c+1/d)^0.5^ |  |
| **PRR** | PRR=(a(c+d))/(c(a+b)) | PRR≥2, X²≥4, N≥3 |
|  | *χ2*=[(ad-bc)^2](a+b+c+d)/[(a+b)(c+d)(a+c)(b+d)] |  |
| **BCPNN** | IC=log_2_a(a+b+c+d)(a+c)(a+b) | IC_025_>0 |
|  | IC_025_=e ^ln(IC)-1.96(1/a+1/b+1/c+1/d)^0.5^ |  |
| **MGPS** | EBGM=a(a+b+c+d)/((a+c)/(a+b)) | EBGM05>2, N>0 |
|  | EBGM05=e ^ln(EBGM)-1.64(1/a+1/b+1/c+1/d)^0.5^ |  |

*ROR*, reporting odds ratio; *PRR*, proportional reporting ratio; *EBGM*, empirical Bayes geometric mean; *IC*, information component; *CI*, confidence interval; *95% CI*, two-sided for ROR; *χ2*, chi-squared; *EBGM05 and IC025*, lower one-sided for EBGM and IC, respectively. *a*, the number of reports of osteoporosis events among proton pump inhibitors (PPIs) users; *b*, the number of reports of other adverse events among PPIs users; *c*, the number of reports of osteoporosis events among users of other drugs; *d*, the number of reports of other adverse events among users of other drugs.

**Table S2** Positive signals of five proton pump inhibitors at the SOC level.

| **Drug** | **SOC** | **n** | **ROR** | **Lower** | **Upper** |
| --- | --- | --- | --- | --- | --- |
| **Esomeprazole** | GASTROINTESTINAL DISORDERS | 40422 | 2.24 | 2.21 | 2.26 |
|  | RENAL AND URINARY DISORDERS | 53473 | 16.59 | 16.43 | 16.75 |
|  | METABOLISM AND NUTRITION DISORDERS | 5439 | 1.08 | 1.05 | 1.11 |
|  | MUSCULOSKELETAL AND CONNECTIVE TISSUE DISORDERS | 14847 | 1.21 | 1.19 | 1.23 |
|  | ENDOCRINE DISORDERS | 1925 | 3.28 | 3.13 | 3.43 |
| **Omeprazole** | METABOLISM AND NUTRITION DISORDERS | 10521 | 1.77 | 1.74 | 1.81 |
|  | GASTROINTESTINAL DISORDERS | 39224 | 1.74 | 1.72 | 1.75 |
|  | RENAL AND URINARY DISORDERS | 67791 | 18.07 | 17.91 | 18.23 |
|  | ENDOCRINE DISORDERS | 2443 | 3.48 | 3.34 | 3.62 |
|  | BLOOD AND LYMPHATIC SYSTEM DISORDERS | 5621 | 1.17 | 1.14 | 1.21 |
| **Lansoprazole** | GASTROINTESTINAL DISORDERS | 10807 | 1.18 | 1.15 | 1.2 |
|  | RENAL AND URINARY DISORDERS | 45310 | 39.84 | 39.35 | 40.33 |
|  | METABOLISM AND NUTRITION DISORDERS | 2798 | 1.2 | 1.16 | 1.25 |
|  | SURGICAL AND MEDICAL PROCEDURES | 2097 | 1.43 | 1.37 | 1.5 |
|  | ENDOCRINE DISORDERS | 346 | 1.25 | 1.12 | 1.39 |
| **Rabeprazole** | GASTROINTESTINAL DISORDERS | 1391 | 1.69 | 1.59 | 1.79 |
|  | SKIN AND SUBCUTANEOUS TISSUE DISORDERS | 912 | 1.73 | 1.61 | 1.85 |
|  | METABOLISM AND NUTRITION DISORDERS | 459 | 2.14 | 1.95 | 2.35 |
|  | RENAL AND URINARY DISORDERS | 1376 | 8.31 | 7.85 | 8.8 |
|  | HEPATOBILIARY DISORDERS | 202 | 2.19 | 1.9 | 2.51 |
|  | BLOOD AND LYMPHATIC SYSTEM DISORDERS | 224 | 1.29 | 1.13 | 1.48 |
|  | ENDOCRINE DISORDERS | 59 | 2.28 | 1.77 | 2.95 |
| **Pantoprazole** | GASTROINTESTINAL DISORDERS | 12943 | 1.51 | 1.49 | 1.54 |
|  | IMMUNE SYSTEM DISORDERS | 1346 | 1.16 | 1.1 | 1.23 |
|  | RENAL AND URINARY DISORDERS | 20991 | 13.67 | 13.47 | 13.88 |
|  | BLOOD AND LYMPHATIC SYSTEM DISORDERS | 2593 | 1.47 | 1.41 | 1.53 |
|  | METABOLISM AND NUTRITION DISORDERS | 4430 | 2.02 | 1.96 | 2.08 |
|  | HEPATOBILIARY DISORDERS | 1844 | 1.95 | 1.86 | 2.04 |
|  | ENDOCRINE DISORDERS | 524 | 1.98 | 1.82 | 2.16 |
